# Development of Monoclonal Antibody to Specifically Recognize VP0 but Not VP4 and VP2 of Foot-and-Mouth Disease Virus

**DOI:** 10.3390/pathogens11121493

**Published:** 2022-12-08

**Authors:** Sun Young Park, Jong Sook Jin, Dohyun Kim, Jae Young Kim, Sang Hyun Park, Jong-Hyeon Park, Choi-Kyu Park, Young-Joon Ko

**Affiliations:** 1 Center for FMD Vaccine Research, Animal and Plant Quarantine Agency, 177 Hyeoksin-8-ro, Gimcheon-si 39660, Republic of Korea; 2 College of Veterinary Medicine & Animal Disease Intervention Center, Kyungpook National University, Daegu 41566, Republic of Korea

**Keywords:** foot-and-mouth disease virus, VP0, VP2, VP4, monoclonal antibody

## Abstract

Foot-and-mouth disease (FMD) is a highly contagious vesicular disease that affects cloven-hoofed animals and often causes enormous economic loss in the livestock industry. The capsid of FMD virus (FMDV) consists of four structural proteins. Initially, one copy each of the proteins VP0, VP3, and VP1 are folded together into a protomer, and five copies of the protomer compose a pentamer. Finally, 12 pentamers are assembled into an icosahedral capsid. At the maturation stage during RNA encapsidation, VP0 is cleaved into VP4 and VP2. The mechanism underlying VP0 maturation remains unclear. While monoclonal antibodies (mAbs) against VP2 have been developed in previous studies, a mAb specific to VP0 has not yet been reported. In this study, we generated VP0-specific mAbs by immunizing mice with peptides spanning the C-terminal amino acids of VP4 and N-terminal amino acids of VP2. We verified that these mAbs displayed specificity to VP0 with no reactivity to VP4 or VP2. Therefore, these mAbs could prove useful in identifying the role of VP0 in FMDV replication and elucidating the mechanism underlying VP0 cleavage into VP4 and VP2.

## 1. Introduction

Foot-and-mouth disease (FMD) is a clinically severe acute vesicular disease in cloven-hoofed animals, including ruminants and pigs [1]. The causative FMD virus (FMDV) consists of a single-stranded plus-sense RNA genome of approximately 8500 bases surrounded by four structural proteins. FMDV belongs to the genus *Aphthovirus* of the *Picornaviridae* family. Seven serotypes (O, A, Asia 1, C, and South African territories 1, 2, and 3) have been identified serologically, and multiple subtypes occur within each serotype [2].

Sixty copies of each of the four structural proteins (VP4, VP2, VP3, and VP1) are assembled in a stepwise process to form the viral capsid. Initially, one copy each of the proteins VP0, VP3, and VP1 are folded together into a protomer, and five copies of the protomer compose a pentamer. Ultimately, 12 pentamers are assembled into an oligomeric protein shell [3]. The assembly of the viral capsid and the encapsidation of the viral RNA involve the cleavage of the VP0 precursor into VP2 and VP4. However, the specific cleavage mechanism remains unclear [4,5]. VP1, VP2, and VP3 are surface-exposed, each adopting an eight-stranded beta-barrel conformation with extended N- and C-termini [6]. VP4 is internal and varies in structure and position among different picornaviruses.

In addition to its structural role in the process of virus assembly, VP0 also interacts with poly (rC)-binding protein 2 (PCBP2), increasing the PCBP2-mediated degradation of VISA (CARD-containing adaptor, also known as MAVS, IPS-1, or Cardif) and subsequently increasing FMDV replication [7].

To elucidate the exact role of VP0 in the process of FMDV replication and understand the mechanism underlying the maturation and cleavage of VP0 into VP4 and VP2, a monoclonal antibody (mAb) specific to VP0 is needed. Although mAbs against VP2 are available [8], research on the production of mAbs against VP0 has not been widely reported. Therefore, this study aimed to generate mAbs that react specifically with VP0 and not with VP2 and VP4.

## 2. Materials and Methods

### 2.1. Peptide Synthesis

**Figure 1:**
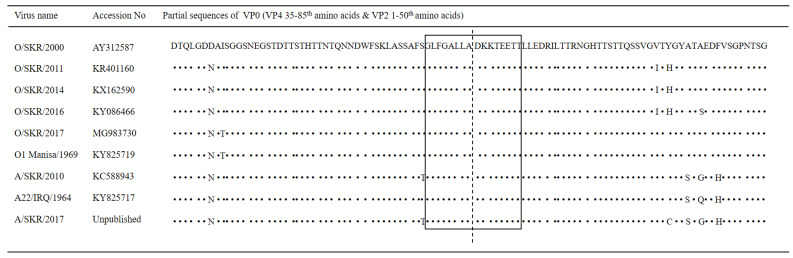
Alignment of the amino acid sequences in the junction region between VP4 and VP2 of different strains of foot-and-mouth disease virus (FMDV). The amino acid sequences of the VP4 C-terminus and VP2 N-terminus derived from seven local strains and two representative strains (O1 Manisa and A22 IRQ) of FMDV were aligned. The box with solid lines indicates the selected sequence (16-mer) for the production of VP0-specific mAb. The sequences of VP4 and VP2 are separated by the dotted line. Dots represent identical amino acids.

A 16-mer peptide (GLFGALLADKKTEETT) spanning 8 amino acids on the C-terminal of VP4 and 8 amino acids on the N-terminal of VP2 was synthesized by Anygen (Gwangju, Republic of Korea) based on the sequences of FMDV O/Boeun/SKR/2017 (GenBank Accession No. MG983730.1), as shown in Figure 1. One cysteine residue was added to the N-terminal amino acid to facilitate carrier conjugation. The peptides, which would function as the immunogen for the production of mAbs and as antigens for enzyme-linked immunosorbent assay (ELISA) screening, were conjugated using keyhole limpet hemocyanin (KLH) and bovine serum albumin (BSA), respectively. The purity of the synthesized peptide was determined to be >80% using high performance liquid chromatography.

### 2.2. Production of mAbs

BALB/c mice were immunized subcutaneously twice with a KLP-conjugated 16-mer peptide corresponding to the VP0 epitope. Hybridoma cells were generated by the fusion of murine lymphoid cells with the myeloma cell line and screened using peptide ELISA. The ovalbumin-conjugated VP0 epitope peptide (250 ng/well) was coated onto plates with 0.1 M bicarbonate buffer (pH 9.6) and incubated overnight at 4 °C. After discarding the coating solution, 200 µL of blocking solution (2% skimmed milk in phosphate-buffered saline (PBS)) was added to the plates and incubated at 37 °C for 1 h. After washing once with Tris-buffered saline with Tween-20 (TBST), 50 µL of primary antibody (cell culture supernatant) was added to the plates and incubated at 37 °C for 2 h. After washing thrice with TBST, 50 µL of horseradish peroxidase (HRP)–labeled goat anti-mouse IgG antibodies (Thermo Fisher Scientific, Waltham, MA, USA) were added at a ratio of 1:10,000, and the plates were incubated at 37 °C for 1 h. The plates were washed five times with TBST, and 50 µL 3,3′,5,5′-tetramethylbenzidine (TMB) substrate was added (Surmodics, MN, USA). When the color changed to blue, a stop solution (1N H_2_SO_4_) was added to the plates, and the optical density was measured at 450 nm. Finally, the isotypes of the selected mAbs were identified using the Rapid ELISA Mouse mAb Isotyping Kit (Thermo Fisher Scientific, Waltham, MA, USA). Animal experiments in this study were approved by the Institutional Animal Care and Use Committee (AEC-20081204-0001).

### 2.3. Cloning and Expression of Recombinant Proteins

The DNA fragment of the FMDV VP0 epitope (16-mer) was cloned into the pGEX-4T-1 vector (GE Healthcare, Piscataway, NJ, USA) by Enzynomics (Daejeon, Republic of Korea) to construct recombinant expression plasmids designated as glutathione S-transferase (GST)–fused VP0 epitopes. The recombinant protein expression of the GST-VP0 epitope was carried out according to the manufacturer’s instructions. Briefly, the recombinant plasmid was transformed into the *Escherichia coli* (*E. coli*) BL21 (DE3) strain, and the cells were cultured in Luria–Bertani medium containing 50 μg/mL kanamycin at 37 °C until the optical density reached 0.6 at 600 nm. The expression of recombinant proteins was induced by adding 1 mM isopropyl-β-d-1-thiogalactopyranoside at 37 °C for 4 h. Pellets were harvested from the culture by centrifugation at 3000× *g* for 10 min, resuspended in lysis buffer (50 mM Na_2_HPO_4_ and 300 mM NaCl, pH 8.0), and lysed by sonication (20 cycles, pulse on-time 5 s, and off-time 10 s at 20% amplitude). The cell lysate was centrifuged at 3000× *g* for 10 min, and the soluble protein fractions were collected and purified using affinity column chromatography with glutathione sepharose 4 B resin (GE Healthcare) according to the manufacturer’s instructions. 

The coding sequences of VP0 (909 bp), VP4 (255 bp), and VP2 (654 bp) were synthesized by Bioneer (Daejeon, Republic of Korea). The DNA fragments of VP0, VP4, and VP2 were cloned into the pGEX-4T-1 vector (GE Healthcare) to construct recombinant expression plasmids designated as GST-VP0, GST-VP4, and GST-VP2, respectively. The expression and purification procedures for these GST-fused proteins were performed in the same manner as that for the GST-VP0 epitope.

### 2.4. SDS-PAGE and Western Blot Analysis

Protein samples were subjected to sodium dodecyl sulfate-polyacrylamide gel electrophoresis (SDS-PAGE) and Western blot analysis. The experimental procedures were performed according to the protocol of the previous study [9]. The following antibodies were used in Western blot analysis: anti-FMDV VP0, anti-FMDV VP4 (Median Diagnostics, Chuncheon, Republic of Korea), anti-FMDV VP2 (Median Diagnostics), and anti-GST (Invitrogen) antibodies. For anti-FMDV VP0, the hybridoma cell supernatant was used, and other antibodies were diluted 2000-fold in PBS. 

## 3. Results

### 3.1. Selection of Amino Acid Sequence for the Production of mAb against FMDV VP0

To produce mAb against VP0 of FMDV, the amino acid sequences at the VP4 C-terminal and VP2 N-terminal were aligned (Figure 1). The sequences were derived from FMDV isolated during FMD outbreaks in South Korea (2000, 2011, 2014, 2016, and 2017), as well as from two representative strains, O1 Manisa and A22 IRQ. These sequences (the C-terminal amino acids of VP4 and N-terminal amino acids of VP2) are conserved among several FMDV strains of serotypes O and A. Therefore, two sequences comprising eight amino acid residues from VP4 and eight amino acid residues from VP2 (GLFGALLADKKTEETT) were selected for the production of mAb.

### 3.2. Production of FMDV VP0-Specific mAbs

Peptides were synthesized using amino acid sequences (16-mer) spanning VP4 and VP2, as shown in Figure 1. After immunization of the mice with the peptide conjugated with KLH, the hybridoma supernatant was screened using ELISA with the BSA-conjugated VP0 epitope peptide as an antigen. Five hybridoma clones producing the FMDV VP0 antibody were selected and are shown in Table 1. The optical density measured using peptide ELISA was approximately 2.0 for all five hybridoma supernatants. All the mAbs consisted of either IgG1 or IgG2b heavy chains and kappa light chains.

### 3.3. Cloning and Expression of Recombinant Proteins

**Figure 2:**
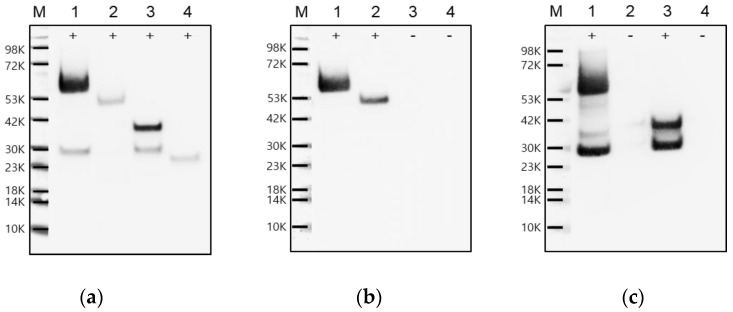
Identification of the recombinant FMDV VP0, VP2, VP4, and VP0 epitope proteins. The glutathione S-transferase (GST)–fused recombinant proteins were each expressed in *Escherichia coli* and detected using Western blot with (**a**) anti-GST, (**b**) anti-VP2, and (**c**) anti-VP4 antibodies. Lane M, molecular weight protein marker; lane 1, GST-VP0 protein; lane 2, GST-VP2 protein; lane 3, GST-VP4 protein; lane 4, GST-VP0 epitope protein.

To evaluate the reactivity of the VP0-specific mAbs, the DNA fragments corresponding to the peptides (16-mer) used for mAb production were cloned into the pGEX-4T-1 vector to generate a plasmid for GST-fused recombinant VP0 epitope protein expression. The genes corresponding to VP4, VP2, and VP0 of FMDV O/Boeun/SKR/2017 were also cloned into the same vector. Each of the four recombinant proteins was expressed in *E. coli*. As shown in Figure 2, VP0, VP2, VP4, and VP0 epitopes were identified as recombinant proteins fused to GST using an anti-GST antibody in Western blot analysis; the molecular weights were detected to be 60, 51, 36, and 26 kDa, respectively. Whereas the anti-VP2 antibody identified VP2 and VP0, the anti-VP4 antibody identified VP4 and VP0. The VP0 epitope showed no reactivity with either anti-VP2 or anti-VP4 antibodies. An additional small band of approximately 30 kDa was detected in the GST-VP0 and GST-VP4 lanes (Figure 2a,c). This is assumed to be a fusion protein of GST and a partially soluble region at the VP4 N-terminus—possibly formed by cleavage at the N-terminal sequences of VP4 for unknown reasons. Previously, it has been reported that Triatoma picornavirus VP4 is presumed to cause apoptosis induction [10]. Therefore, we suspect that this cleavage may be related to the cytotoxicity of VP4.

### 3.4. Reactivity of mAbs with Recombinant Proteins

**Figure 3:**
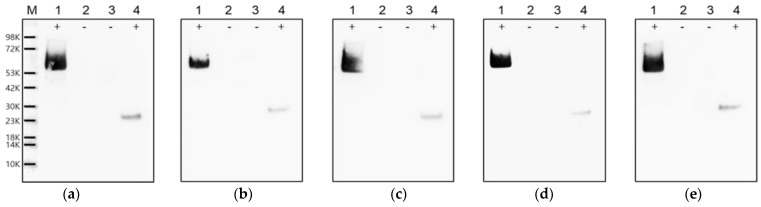
Reactivity of the FMDV VP0-specific monoclonal antibodies (mAbs) with the recombinant proteins. The reactivity of five types of VP0-specific antibodies, (**a**) 8G9, (**b**) 9A5, (**c**) 17E6, (**d**) 23F2, and (**e**) 24A12, to the GST-fused recombinant VP0, VP2, VP4, and VP0 epitope proteins was evaluated using Western blotting. Lane M, molecular weight protein marker; lane 1, GST-VP0 protein; lane 2, GST-VP2 protein; lane 3, GST-VP4 protein; and lane 4, GST-VP0 epitope protein.

Western blot analysis of the mAbs was performed with the previously expressed recombinant proteins to confirm whether the five mAbs selected using peptide ELISA were specific to the VP0 epitope region. As shown in Figure 3, all mAbs reacted with the GST-VP0 epitope and GST-VP0 recombinant proteins, but they did not react with the GST-VP2 or GST-VP4 recombinant proteins.

## 4. Discussion

The structural proteins VP0, VP1, and VP3 of FMDV are synthesized during translation in the cell at the proliferation stage to form protomers, and five protomers are assembled into a pentamer. Twelve pentamers are incorporated into the immature FMDV particle. During the final maturation stage, RNA enters the particle, and concurrently, VP0 is cleaved into VP4 and VP2 [11]. This phenomenon is common to most picornaviruses, including FMDV; however, the exact mechanism is not yet known.

VP4 and RNA are released from the viral particle when FMDV invades cells or due to the low pH in the endosome or on exposure to high temperature in the external environment. In other words, VP0 is an intermediate structure that appears transiently during virus particle formation [12]. Since myristate (a fatty acid) is bound to the glycine residue at the N-terminus of VP4 and this fatty acid conjugation affects VP0 cleavage, a viral inhibitor that prevents the proliferation of picornaviruses by blocking the myristoylation of VP4 has also been reported [13,14,15].

Recently, VP0 has been reported to interact with intermediates involved in interferon-related signal transduction, disturbing the cellular immune system and leading to viral replication [7].

Although VP0 plays various pivotal roles in structural arrangement and cellular physiology, these studies were conducted using antibodies against VP2, which detected both VP0 and VP2. Hence, an antibody that specifically binds to VP0 is essential to accurately analyze the cellular function of VP0 and its reactivity with virus particles before and after cleavage into VP2 and VP4. However, studies focusing on the development of an antibody that exclusively binds to VP0 have not been undertaken. In this regard, this study aimed to produce a mAb that specifically binds to VP0, but not to VP2 or VP4.

VP4 is the most conserved protein among the seven serotypes of FMDV, and the N-terminal region of VP2 is highly conserved [16,17]. Therefore, much research has been carried out to develop methods for the detection of antibodies against all serotypes using the conserved regions of VP4 or VP2 [18] or to develop techniques for the detection of all serotypes of FMDV using mAbs against VP2 [8,19,20,21].

As shown in Table 1, the amino acid sequences of the VP4 C-terminus and VP2 N-terminus were identical in many strains of FMDV serotypes O and A in addition to being conserved in all seven serotypes of FMDV [20]. A previous study reported an epitope capable of detecting all FMDV serotypes located in the N-terminal region of VP2 [8]. 

Therefore, we synthesized a 16-mer peptide spanning eight amino acids at the VP4 C-terminus and eight amino acids at the VP2 N-terminus to produce a mAb that specifically binds to VP0. The number of amino acid residues was determined based on a previous report that peptides used for immunization are usually 10–20 amino acids long; peptides less than 10 amino acids or longer than 20 amino acids may elicit antibodies that do not recognize the protein with sufficient affinity or specificity and are hence not preferred [22].

In this study, we successfully produced five FMDV VP0-specific mAbs after immunizing mice with the synthesized 16-mer peptide, as previously described (Table 1). Moreover, all five mAbs were reactive to both recombinant GST-VP0 and GST-VP0 epitope proteins containing the selected amino acid sequence (16-mer) by Western blot analysis, demonstrating the reactivity with the VP0 epitope (Figure 3).

Based on a previous report that major epitopes were located at residues 8–18 and 28–40 of VP4 and the N-terminus of VP2, we initially anticipated that most mAbs produced in mice would be exclusive against VP2 or VP4 [23]. In addition, one group of researchers tried to synthesize mAbs against VP0 by immunizing mice with VP0 of human parechovirus; however, the resulting mAb recognized only the VP2 region and was not specific to VP0 [24]. In contrast, we successfully generated mAbs that reacted only to VP0 and not to VP4 or VP2, making this the first report on the production of mAbs specific to the VP0 of FMDV.

Furthermore, this strategy of generating mAbs using overlapping sequences for unknown epitopes could shed light on the development of a variety of novel mAbs based on the junction regions between two different proteins’ subunit in viruses.

## 5. Conclusions

This is the first report of a mAb that specifically recognizes the VP0 of FMDV. The mAbs developed in this study could be used to elucidate the role of VP0 in FMDV replication and the mechanism underlying the cleavage of VP0 into VP4 and VP2.

## Figures and Tables

**Table 1:** Characterization of the monoclonal antibodies against VP0 produced in mice.

Clone	Optical Density (ELISA) *	Heavy Chain	Light Chain
8G9	1.925	IgG2b	Kappa
9A5	2.033	IgG1	Kappa
17E6	1.952	IgG1	Kappa
23F2	1.953	IgG1	Kappa
24A12	2.161	IgG2b	kappa

* ELISA using bovine serum albumin–conjugated peptide composed of 16 amino acids as coating antigen. The values were all background-subtracted (absorbance—average blank).

## Data Availability

Not applicable.
